# Photo-flocculation of microbial mat extracellular polymeric substances and their transformation into transparent exopolymer particles: Chemical and spectroscopic evidences

**DOI:** 10.1038/s41598-017-09066-8

**Published:** 2017-08-22

**Authors:** Mashura Shammi, Xiangliang Pan, Khan M. G. Mostofa, Daoyong Zhang, Cong-Qiang Liu

**Affiliations:** 1 Laboratory of Bioremediation, Department of Environmental Pollution and Process Control, Xinjiang Institute of Ecology and Geography, Chinese Academy of Sciences, Urumqi-830011, Xinjiang, P.R. China; 2 Department of Environmental Sciences, Jahangirnagar University, Dhaka, 1342 Bangladesh; 3 Institute of Surface-Earth System Science, Tianjin University, 92 Weijin Road, Nankai District, Tianjin, 300072 P.R. China; 4 State Key Laboratory of Environmental Geochemistry, Institute of Geochemistry, Chinese Academy of Sciences, Guiyang, 550002 Guizhou P.R. China; 5 College of Environment, Zhejiang University of Technology, Hangzhou, 310014 Zhejiang, P.R. China; 6 University of Chinese Academy of Sciences, Beijing, 100049 P.R. China

## Abstract

Upon exposure to sunlight extracellular polymeric substances (EPS) were partially transformed into transparent exopolymer particles (TEP) and unstable flocs of different sizes without the addition of any precursors. Parallel factor (PARAFAC) modelling of the sample fluorescence spectra identified humic-like and protein-like or tyrosine-like components in both untreated and irradiated EPS samples. After 58 hours of solar irradiation, humic-like substances were entirely decomposed, while the regenerated protein-like substance from EPS was the key component in the irradiated samples. Degradation and reformation of EPS occurred which was confirmed by the results of size exclusion chromatography, dissolved organic carbon, total protein and total polysaccharide analyses. Irradiated EPS was composed of –COOH or C = O (amide I band) and –NH and –CN (amide II band), while Fourier transform infrared spectroscopy (FTIR) of TEP revealed more acidic –COOH and –C–O groups, indicating typical acidic protein-like TEP. The regenerated protein-like substances could form complexes with free metals originating from degraded EPS in irradiated samples, which could be responsible for the formation of TEP/floc in the aqueous media. These results suggest that TEP/floc formation from EPS could occur by a complexation mechanism between dissolved organic matter and metals, thereby causing ionic charge neutralisation upon sunlight exposure.

## Introduction

Bacterial biofilms are formed by communities that are embedded in a self-produced matrix of extracellular polymeric substances (EPS)^1^, which is a term encompassing a large group of very different biopolymers. The biofilm matrix can be considered an external property of the microorganisms, allowing them to form stable synergistic consortia, supporting interaction with signalling molecules and horizontal gene transfer and, eventually, activation by extracellular enzymes, which turn the matrix into an external digestive system^2^. EPS are a high-molecular-weight (MW > 410,000) mixture of polymers that are composed mainly of polysaccharides, proteins, nucleic acids, lipids, surfactants and humic-like substances^3, 4^. Humic substances are the integral part of the EPS^3–5^, which can participate in complexation of trace elementsand random flocculation in natural water environments^6–9^. Therefore, EPS composition can be variable which determines its reactivity and structural function^10^. They provide the mechanical stability of biofilms, mediate their adhesion to surfaces and form a cohesive, three-dimensional polymer network that interconnects and transiently immobilises biofilm cells^3^. EPS enhances resistance to stress caused by toxicity or environmental variables^10^. Biogeochemical cycling of elements- particularly, heavy metals in different chemical forms, mobility, bioavailability and ecotoxicity are significantly influenced by EPS in the aquatic environment^6^. Furthermore, in EPS-dominated biofilm systems, interactions involve both surface complexation to EPS/cells and mineral products of metabolic/abiotic processes^10^.

Transparent exopolymeric particles (TEP) are operationally defined as larger than 0.4 μm, whereas the other substances chemically identical to TEP, but smaller than 0.4 μm, can be considered as TEP precursors^11^. TEP and their precursors are considered as a “planktonic” subgroup of EPS or hydrogel subgroups because they originate via the release of extracellular, acidic polysaccharides produced by phyto-/bacterioplankton^11, 12^. TEP are polysaccharide particles, formed by the aggregation of polymers exuded by phytoplankton and are strongly involved in organic matter sedimentation^13^. Frequently, TEP are intensely colonised by bacteria and other microorganisms, thus serving as hot spots of intense microbial activity and biofilm formation^14^. TEP and other microgel particles in marine and freshwaters are part of a size continuum of organic matter that includes polymers, nanogels, microgels, and very large marine (or lake) snow particles (macrogels)^14^ that are significant and critical in sedimentation processes^15^.

Flocculation is extensively employed for clarification through sedimentation in water treatment works^16^. Flocculation has major implications in organic matter (OM) transformation and removal pathways^7^ in aquatic environments. Without chemical reactions dissolved organic matter (DOM) could spontaneously entangle to form particulate organic matter (POM) microgels^17^ and correspondingly, photochemical flocculation of terrestrial DOM and Fe^8^. Oftentimes, microbial ferrous iron [Fe(II)] oxidation leads to the formation of iron-rich macroscopic aggregates (“iron snow”) of ferric iron [Fe(III)] in the surface waters depending on surrounding geochemistry^18^. Conversely, to understand the kinetics of OM cycling in aquatic environments, it is crucial to achieve a mechanistic and molecular understanding of its transformation processes^19^. EPS was transformed into unstable flocs by UV (ultraviolet) radiation and stable flocs by simulated solar radiation of 70 μWcm^−2^ (irradiation time 120 min)^20^. However, the study solely identified turbidity as a precursor to floc formation and many important characteristics behind the flocculation process, i.e., particle size, surface charges, DOC changes and fluorescent component changes were overlooked. Therefore, it is vital to identify physical, chemical and fluorescent characteristics during the transformation processes of EPS to TEP/flocs during natural sunlight exposure.

However, the link between photoinduced alteration of EPS and its TEP/floc formation behaviour has received relatively little attention. Therefore, to elucidate the effect of sunlight on EPS photo-flocculation and TEP formation, an experiment was designed to study the formation of TEP from 0.22-µm filtered fraction of EPS. TEP/floc formation was carried out on treated EPS before and after exposure to sunlight at different intervals across the five consecutive days of irradiation. Conventional chemical and spectroscopic methods were used to characterise the TEP/floc formation along with physicochemical changes in the irradiated EPS samples. New insights into the mechanism of photo-flocculation are comprehensively discussed based on the evidences provided from different physical, chemical and spectroscopic analyses of EPS and TEP under natural sunlight conditions.

## Results

### Photoinduced transformation of EPS to TEP by Alcian blue dye assay

**Figure 1:**
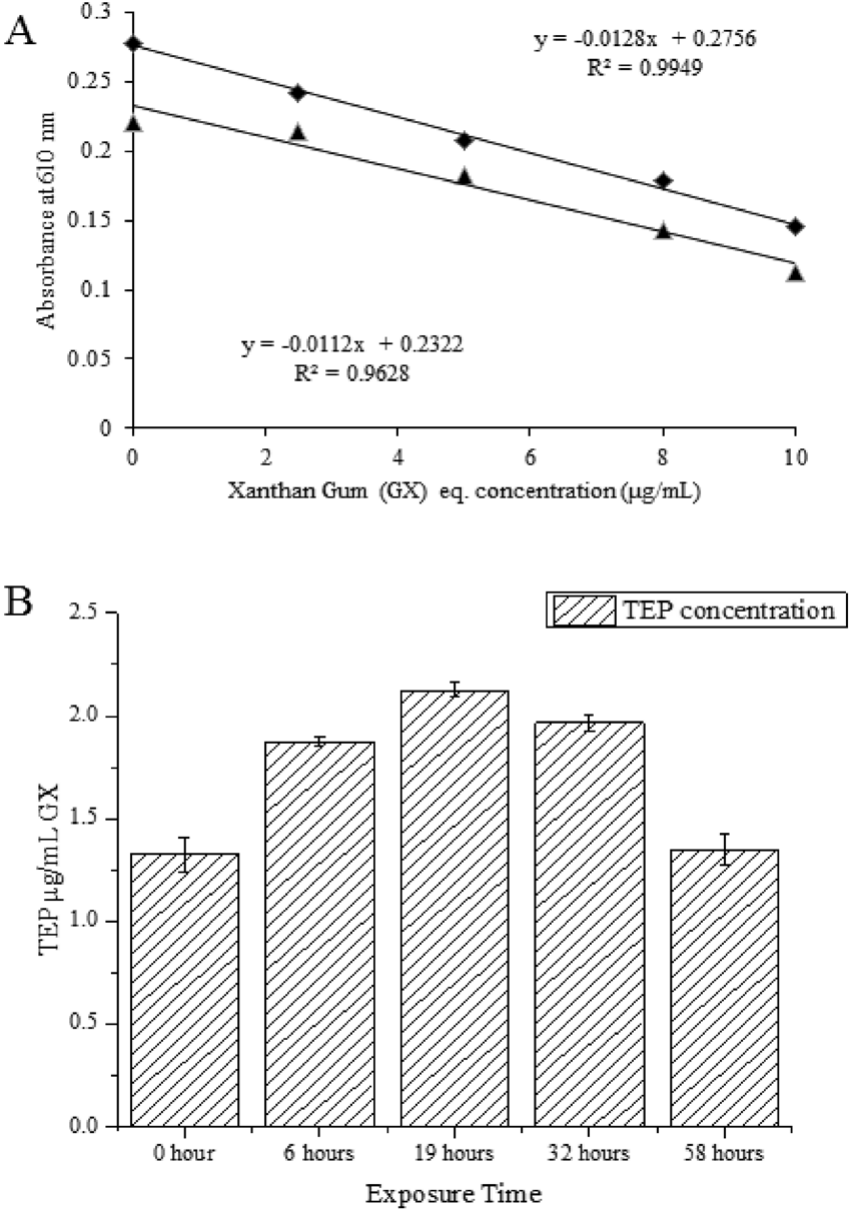
EPS transformation to TEP and flocculation measured as TEP. A. TEP calibration curve with Xanthan Gum; B. concentration of TEP µg/mL GX (equivalent) during photo exposure under light treatment. Only daylight exposure hours (12 hours per day) were counted, to the exclusion of night hours.

**Figure 2:**
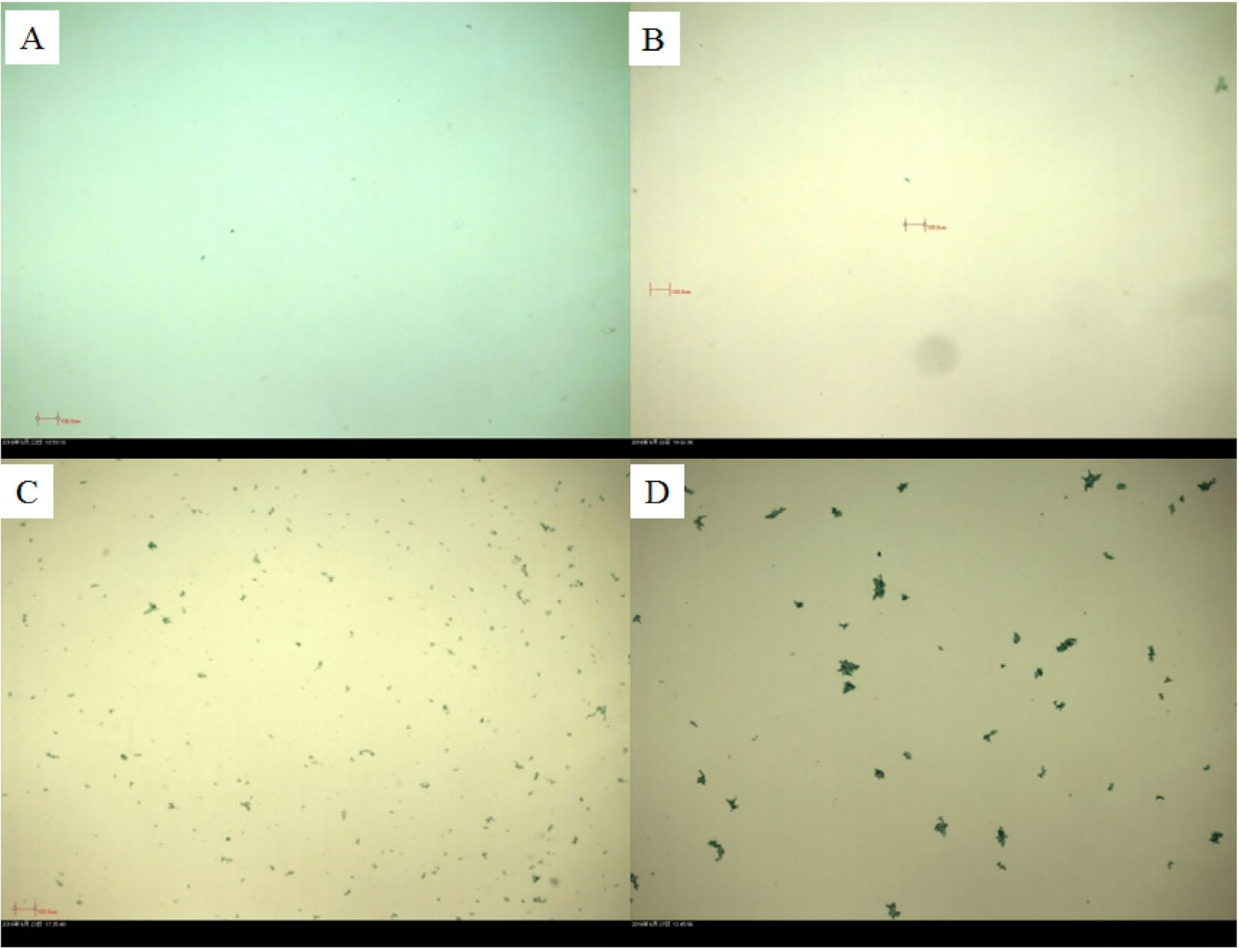
Time-lapse images of EPS to TEP transformation by Alcian Blue dye under sunlight exposures at (**A**) 0 hour, (**B**) 6 hours, (**C**) 19 hours, (**D**) 58 hours. Only daylight exposure hours (12 hours per day) were counted, to the exclusion of night hours. TEP size varied greatly from less than 2 µm to more than 200 µm. All the images were taken at 5× magnification.

After the extraction of EPS using a 0.22-µm filter, the concentration of TEP obtained was approximately 1.32 ± 0.08 µg/mL xanthan gum (GX) equivalent (Fig. 1A,B). After six hours of sunlight irradiation, the TEP concentration had reached 1.87 ± 0.08 µg/mL GX, which was a 41.7% increase compared to the original sample. However, no significant size change was observed from image analysis during this time (Fig. 2). Within 19 hours and 32 hours TEP concentration varied from 1.96 to 2.16 µg/mL GX equivalents, respectively. Consequently, particle size was more visible (Fig. 2). After 58 hours of sunlight exposure TEP concentration had decreased slightly to 1.35 ± 0.07 µg/mL GX equivalents with more visible particles (Fig. 2D). These results imply that EPS transformed into TEP simultaneously during different timescales upon light exposure and that TEP size increased significantly with increasing time (Fig. 2A,D).

### Photo-flocculation of EPS by turbidity, ζ-potential and PSD assay

**Figure 3:**
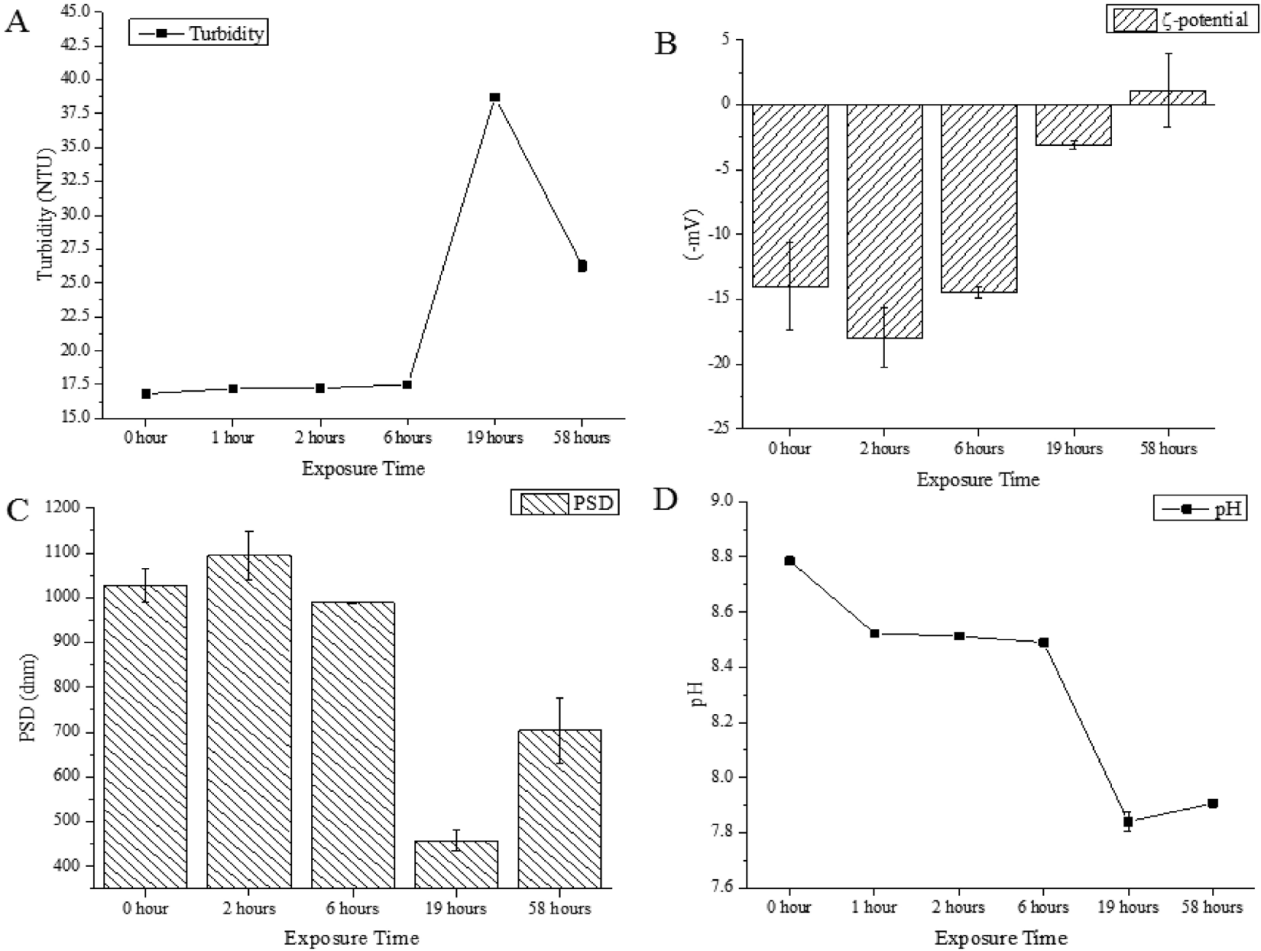
EPS transformation to TEP and flocculation measured as: (**A**) photo-flocculation of EPS upon light exposure, measured as turbidity (NTU); (**B**) ζ−potential (−mV) of light exposure; (**C**) particle size distribution (dnm) of EPS treated in sunlight; (**D**) changes in pH of EPS in light conditions after exposure in sunlight. Only daylight exposure hours (12 hours per day) were counted, to the exclusion of night hours.

Turbidity results showed that a significant increase in turbidity of approximately 56.5% with respect to the initial turbidity of the sample was observed after 32 hours of sunlight exposure (Fig. 1A). However, a 47.3% decrease in turbidity was observed after 58 hours. This result is in accordance with that of a previous study, which reported an increase in turbidity from 11.2 to 22.3 NTU within two hours under simulated solar radiation^20^. It apparently reveals that EPS are decomposed, thereby forming gel-like TEP and floc in aqueous media. Larger floc particles settled on the bottom of the flask, as the turbidity was found to decrease upon light exposure after the 19 hours of exposure. ζ-potential (–mV), the net ionic surface charge of the EPS, varied considerably which indicated the formation of unstable flocs of EPS. However, after the 19 hours of sunlight exposure, ζ-potential decreased to –1.08 (mV), which is approximately a 92% reduction in surface charge compared to the original sample (Fig. 3B). Reducing the magnitude of negative ζ-potential indicates charge neutralisation and destabilisation, which is the well-known conceptual model for the polymer flocculation mechanism^16, 21^.

The role of EPS in the dynamic self-flocculation upon light exposure was observed using particle size distribution (PSD) assay (Fig. 3C). The average PSD of the particles in the extracted EPS was approximately 1000 dnm. The polymers in the EPS self-assembled and flocculated to become particles in natural water. This was further justified by a significant decrease of the surface charge (Fig. 3C), which subsequently caused EPS aggregation in the first 6 hours. Such an effect was finally determined to increase particle size by as much as 33% in the treated samples. This implies that each collision between EPS and/or TEP/floc particle resulted in aggregation that leads to particles sticking together as well as breakage. After 58 hours of sunlight exposure particle size was found to be significantly lower than the original sample by as much as 55.4% (Fig. 3C). This indicates that the polymers of EPS formed flocs by aggregation and disaggregated into smaller particle size of approximately 700 dnm by sunlight irradiation. Previously, it was reported that EPS themselves can aggregate to form particles of about 300 nm, regardless of photic conditions^22^. Furthermore, the UV light intensity which varied from 5–10 Wm^−2^ during the experimental day times might be involved in the formation of unstable polymer flocs, followed by subsequent photolysis of the flocs^17^. The most important factor affecting the flocculation process is pH^23, 24^. During the irradiation time, the pH changed over the course of the five-day experiment. The original pH (8.78) was decreased gradually reaching 7.91 in the irradiated samples (Fig. 3D). Lowering of the pH may be related to the formation of acidic photoproducts^25^. However, the finding is in contrast with that of a previous study^8^ where during the irradiation, pH increased; POM and particulate Fe also formed. Such deviations in results could be caused due to variations in the DOM samples.

### Changes in the fluorescent substances during photo- and microbial flocculation as identified by EEM-PARAFAC modeling

**Figure 4:**
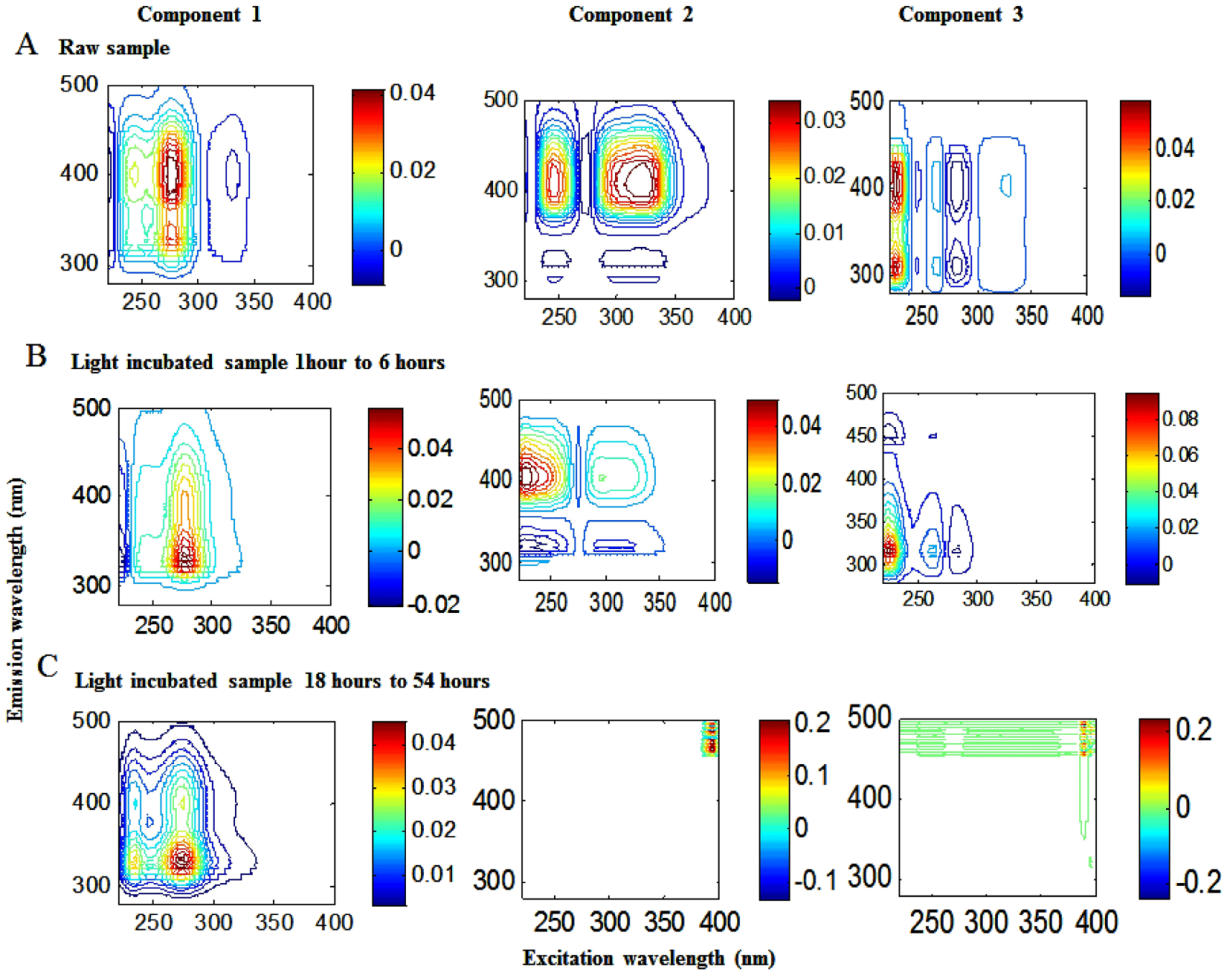
Fluorescent components of EEM-PARAFAC identified by three-component analysis for: (**A**) Raw EPS samples, (**B**) sunlight irradiated EPS sample from 1 hour to 6 hours and (**C**) sunlight irradiated EPS sample from 19 hours to 58 hours of exposure. Only daylight exposure hours (12 hours per day) were counted, to the exclusion of night hours.

The results from the PARAFAC modelling on the raw EPS samples demonstrated that the EPS had three fluorescent components. The first fluorescent component represented a combined humic-like (peak M at Ex/Em = 275/399 nm and peak A at Ex/Em = 245/399 nm) and protein-like (peak T at Ex/Em = 275/328 nm and peak T_UV_ at Ex/Em = 255/328 nm) substances (Fig. 4A: Component 1). The second fluorescent component was identified as consisting of humic-like substances with two fluorescence peaks, including peak M at Ex/Em = 320/407 nm and peak A at Ex/Em = 245/407 nm (Fig. 4A: Component 2). The third fluorescent component consisted of unknown substances with two fluorescent peaks, both showing shorter excitation wavelengths (Ex/Em = 225/310 nm and Ex/Em = 225/399 nm, respectively) (Component 3: Fig. 4A; Table 1). Detection of the combined humic-like and protein-like fluorescent components by EEM-PARAFAC modelling was reported in an earlier study^26^. This could be indicative of the EPS molecular composition, since chemical and spectroscopic studies also generally found EPS to be composed of protein- and humic-like substances^3, 5^ along with polysaccharides. It is apparent that humic-like and protein-like substances, two major backbone of EPS molecular structure, could have similar functional groups (–COOH and –NH_2_)^9^, which might be responsible to interact with each other and produced combined fluorescent peaks.

**Table 1:** Fluorescence excitation/emission (Ex/Em) wavelengths of the raw and treated EPS and the subsequent characteristic peaks of the reference components.

Components	Excitation and emission maxima (% decrease in fluorescence intensity)	Description	Peak region	Corresponding description in the earlier study
Raw EPS sample fluorescence properties in this study				
Component 1^52^	275/328 and	Protein-like	Peak T	280–300/328–356 nm^9^
	245/328		Peak T_UV_	235–250/338–356- nm^9^
	275/399 and	Humic-like	Peak M	260/423–428 nm^29^; 290–330/358–434 nm^9^
	245/399		Peak A	250–260/380–480^29^; 225–260/358–416^9^
Component 2	320/407 and	Humic like	Peak M	260/423–428 nm^29^; 322/407 nm^27^; 290–330/358–416 nm^9^
	245/407		Peak A	250–260/380–480 nm^29^; 225–260/358–416 nm^9^
Component 3^52^	225/399 and	Unknown components	Peak A	This study
	225/260		Peak T_UV_	This study
Photochemical degradation (1 hour to 6 hours) in this study				
Component 1	275/328 and (−68%)	Protein-like	Peak T	280–300/328–356 nm^27^
	245/328–68%		Peak T_UV_	235–250/338–356- nm^27^
Component 2	295/405 and −26%	Humic-like	Peak M	262/380–420 nm^29^; 322/407 nm^27^; 290–330/358–434 nm^9^
	225/405 −26%		Peak A	260/380–460 nm^29^; 225–260/358–416 nm^9^
Component 3	265/260 and (−68%)	Tyrosine-like	Peak T	265–280/293–213 nm including standard tyrosine^30^; 270–280/293–264 nm^27^
	225/260 (−44%)		Peak T_UV_	230/304–307 nm including standard Tyrosine^27^
Photochemical degradation (19 hours to 58 hours) in this study				
Component 1	275/328 (−62%)	Protein-like	Peak T	280–300/328–356 nm^9^
	235/328 (−62%)		Peak T_UV_	235–250/338–356- nm^9^
Component 2	295/474	Unknown photo-products	Peak C	No reference
Component 3	390/272	Unknown photo-products	Peak C	No reference

(+) and (−): an increase and a decrease in fluorescence intensities (%) of various peaks, respectively due to solar and microbial effects.

The percentage changes in the fluorescence intensities of various components are estimated by comparing the treated samples with raw EPS samples (before irradiation). Only daylight exposure hours (12 hours per day) were counted, to the exclusion of night hours.

The humic-like component in the combined component 1 predominantly showed excitation wavelength maxima (Ex = 275 nm) in the shorter wavelength region compared to the component 2 humic-like substances (Ex = 320 nm). This is assumed to be caused by consequential effects of the interaction of two substances (protein-like and humic-like). To distinguish the protein from the aromatic amino acids (e.g., tryptophan or tyrosine), it was previously shown that the protein-like component is detected at higher fluorescence intensity in the longer wavelength (peak T) region compared to that in the shorter wavelength (peak T_UV_) region, while for tyrosine or tryptophan, fluorescence intensity of peak T is higher than peak T_UV_^9^. Component 2 (humic-like) is also termed as “marine humic-like”, because it is often detected in surface waters, particularly in lakes and oceans^9, 27–30^. Component 3 with unknown fluorescent peaks reported in an earlier study^26^, could also be a backbone component originated from diverse EPS molecular compositions.

Pursuant to the short-term irradiation of sunlight (1 to 6 hours), EEM-PARAFAC modelling identified three components: protein-like (component 1: peak T at Ex/Em = 275/328 nm and peak T_UV_ at Ex/Em = 245/328 nm), humic-like (component 2: peak M at Ex/Em = 295/405 nm and peak A at 225/405 nm) and tyrosine-like (component 3: peak T at Ex/Em = 265/310 nm and T_UV_ at Ex/Em = 225/310 nm) substances (Fig. 4B; Table 1). These components are similar to components produced from raw EPS except for component 3, which is confirmed as a tyrosine-like component^9, 27–30^ (Fig. 4B). Over six hours of sunlight exposure, the fluorescence intensities of component 1 and component 2 decreased up to 68% and 26%, respectively (Table 1). Upon continuous long-term irradiation of EPS samples between 19 hours and 58 hours, three-component PARAFAC modelling identified mostly a protein-like component 1 with two peaks (peak T at Ex/Em = 275/328 nm and peak T_UV_ at 235/328 nm) and two unknown components (component 2: Ex/Em = 295/474 nm and component 3: Ex/Em = 390/492 nm), which were thought to be photo-products from decomposed humic-like fractions (Fig. 4C; Table 1). The fluorescence intensity of protein-like substances (component 1) was reduced by approximately 62% after 58-hours of irradiation. Fluorescence intensity decreased either by TEP/floc formation through complexation between protein-like substances and trace metals, or through its direct mineralisation into other components. This decrease in fluorescence intensity utterly differs from previous report of stable fluorescence^20^; presumably due to the application of low-intensity UV. These results further suggest that protein-like substances predominantly remain until the 58 hours of irradiation, which could play an important role in the photo-flocculation processes.

### Chemical and molecular-level changes of EPS during photo-flocculation

**Figure 5:**
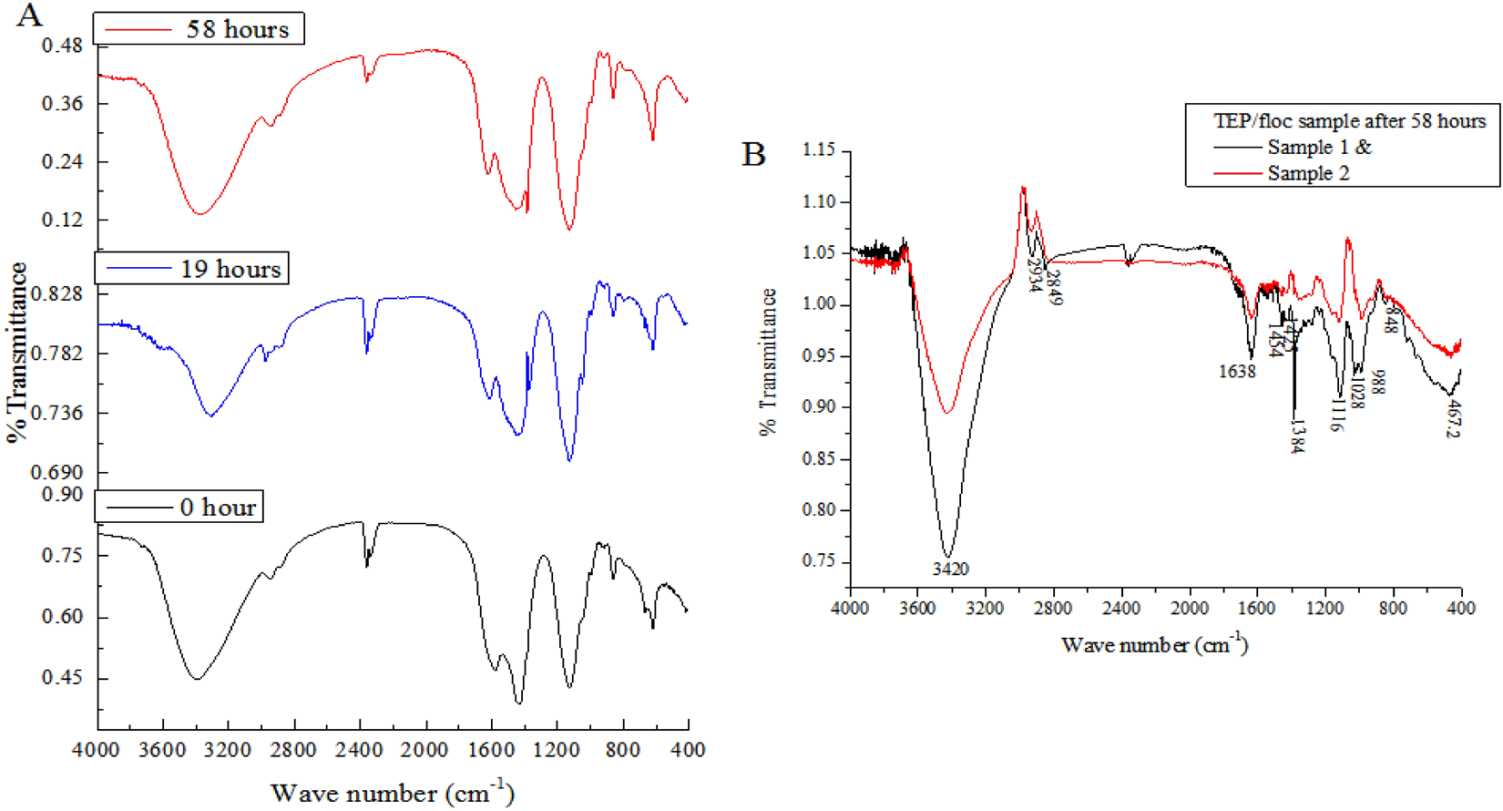
(**A**) FTIR spectrum of EPS with photo exposure at 0 hour, 19 hours and 58 hours; (**B**) FTIR spectrum of TEP/floc filtered on 0.22 µm filter after 58 hours of photo exposure. Only daylight exposure hours (12 hours per day) were counted, to the exclusion of night hours. It is evident from the two figures that compared to the original and light exposed EPS; TEP/flocs collected on 0.22 µm membrane filter contained more acidic protein-like functional groups.

FTIR analyses of original EPS samples and samples subjected to 58 hours of sunlight irradiation demonstrated that extracted EPS contained several major infrared absorption peaks (Fig. 5A,B). The broad and strong band at approximately 3000 to 3735 cm^−1^ was observed due to the stretching of the OH existing in all polymers. A weak C–H band at approximately 2938 to 2949 cm^−1^ indicates the C–H stretching vibration of methyl and methylene groups. Other major peak regions include 400 to 921 cm^−1^, similar to carbohydrates and polysaccharides consequent to glycosidic linkage. A distinct difference was observed in the peak position at 672 cm^−1^ which was found to disappear for the 32 to 58-hour irradiated sample, indicating breakage of the glycosidic linkage of the polysaccharide^30^. Another major peak at 1124 cm^−1^ observed in irradiated and raw EPS samples may indicate the S = O and C−O−S^31^.

Proteins are shown by the distinct peak positions at 1576−1585 cm^−1^ in raw EPS samples and 1617−1640 cm^−1^ in irradiated EPS samples, which may belong to the amide I band and are assignable to C=O of the −COOH stretching vibration. Amide II is shown by the two bands at 1435 cm^−1^ to 1446 cm^−1^, which are assigned to the N−H deformation and C−N stretching in −CO−NH−. A distinct peak position was observed at 1382 cm^−1^, which appeared after 19 hours of irradiation and remained sharper after 58 hours. This indicates the presence of symmetrical stretching of C=O in a –COO^−^ group^32^. Moreover, the FTIR spectrum of 58-hour irradiated TEP/floc which was collected on the 0.22-µm membrane, revealed that, unlike the original EPS, the TEP/floc was characterised by more acidic protein-like components containing −COOH or C=O groups assigned to 1638 cm^−1^ and 1384 cm^−1^ (Fig. 5B). It is well known that amide linkages generally constitute a defining molecular structural feature of proteins. Alcian blue dyes stain the carboxyl and sulphate half-ester groups of the acidic polymers^33^. Moreover, photochemically flocculated POM was enriched in amide functionality^8^. These results therefore imply that TEP/floc could have been formed from protein-like components originating from the EPS upon light exposure.

**Figure 6:**
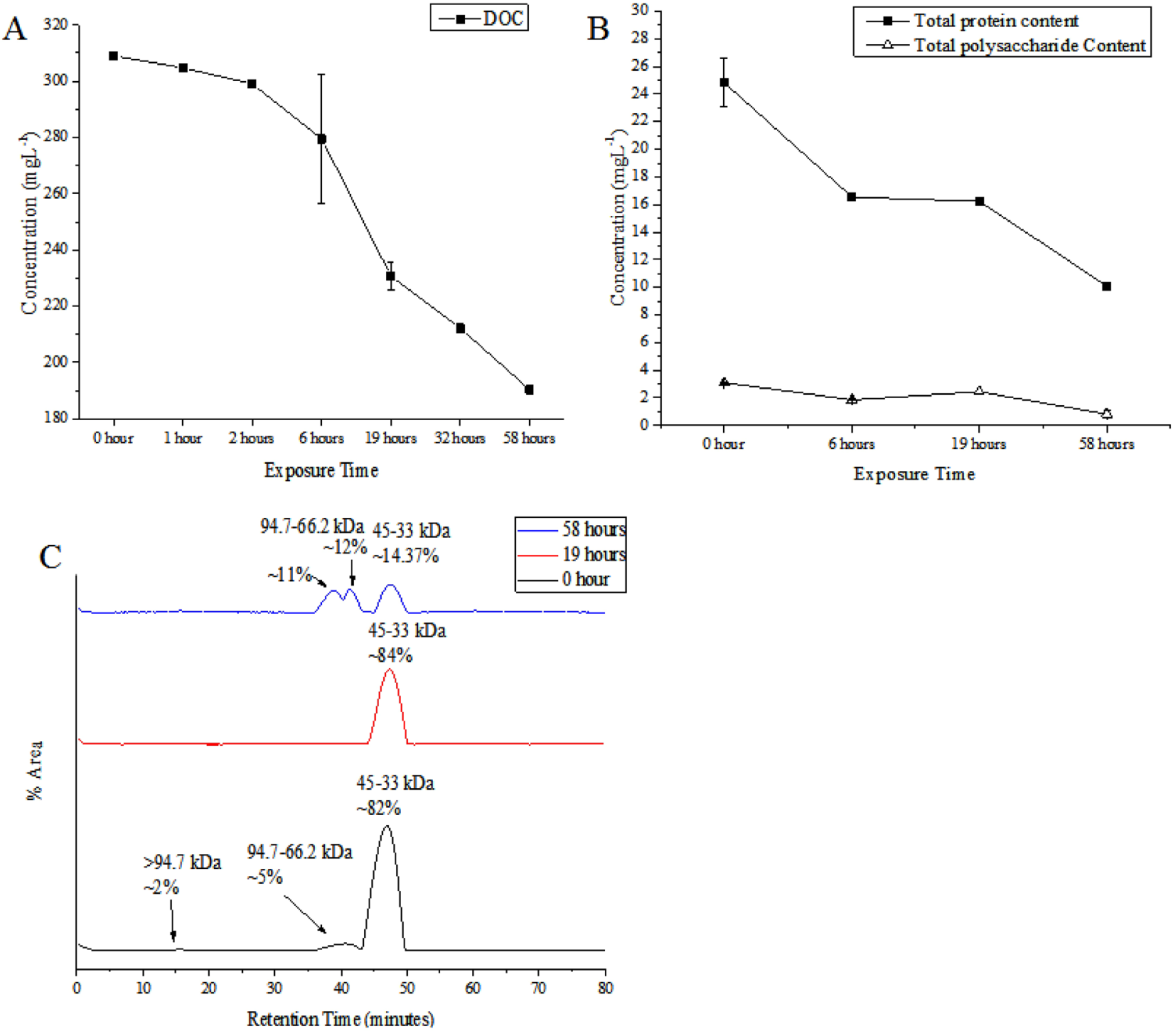
Influences of sunlight on photo-degradation of (**A**) DOC mgL^−1^ content of EPS under light condition; (**B**) changes in total protein and total polysaccharide concentration during the exposure time and (**C**) Fingerprints of EPS protein molecular weight regions at 0 hour, 19 hours and 58 hours. Only daylight exposure hours (12 hours per day) were counted, to the exclusion of night hours.

During the sunlight irradiation and flocculation, dissolved organic carbon (DOC), total protein and total polysaccharide reduction further confirmed mineralisation of the EPS (Fig. 6A,B). A small decrease in the DOC concentration was detected during the first six hours (~9.5%) of irradiation compared to the original DOC (308.97 ± 1.2 mgL^−1^). This decreasing trend was substantially higher as irradiation time increased and ultimately, a 38.4% decrease was detected after 58 hours of irradiation (Fig. 6A). The degradation of DOC was reported in previous studies^9, 34^ and that 7% of the terrestrial DOM of DOC was converted to POC, while 75% was remineralised^8^. A 59% reduction in total protein and a 73% reduction in total polysaccharides were observed after 58 hours of irradiation (Fig. 6B).

To observe changes in protein molecular weight, standard protein and its molecular weight peak were used for the light-treated EPS with respect to the original EPS. According to the retention times and appearances of peaks, four peak positions were considered with the probable molecular weight of >94.7 kDa, 94.7–66.2 kDa, 45–33 kDa and 20–14.4 kDa. The original EPS without sunlight exposure included three distinct peak positions >94.7 kDa, 94.7–66.2 kDa and 45−33 kDa with an area of approximately 2%, 5% and 82%, respectively. After 19-hours of irradiation, the 45–33 kDa peak was found to increase with regard to its percentage area of 84.2%. However, 58-hours of irradiation caused the 94.7–66.2 kDa peak to break into two peaks with approximate areas of 11% and 12%, while the peak of 45–33 kDa had decreased to 14.3% (Fig. 6C). The peaks representative of the high-molecular-weight fractions for the original EPS was decreased in intensities. This result implies a net decrease in molecular weight, which could belong to the low-molecular-weight organic acids^25^. Such changes in the protein-molecular-weights are in agreement with changes in pH, fluorescence, FTIR spectrum and total protein analysis, as discussed earlier, thereby forming TEP/floc.

## Discussion

### A conceptual model of the photoinduced flocculation of EPS

**Figure 7:**
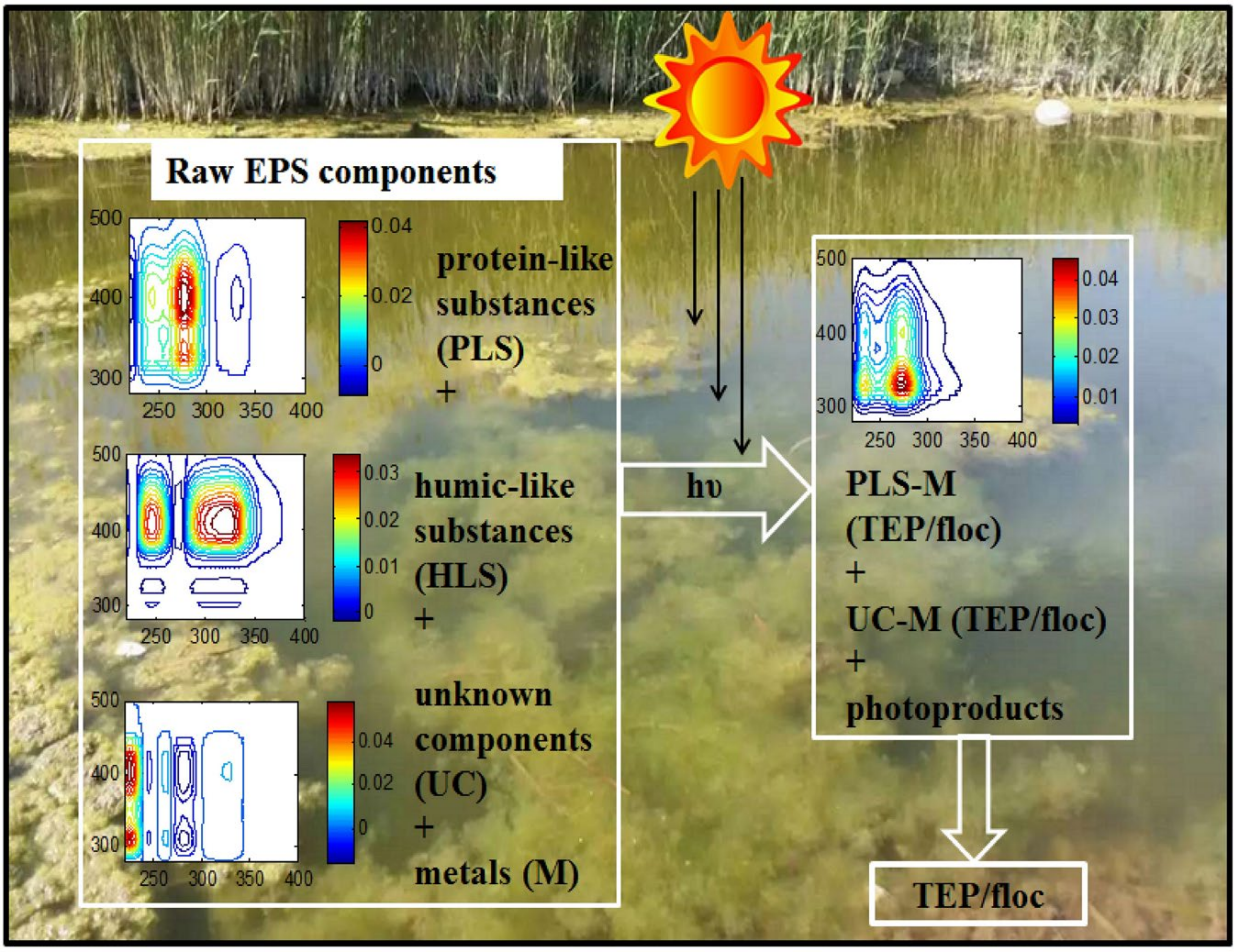
A conceptual model of TEP/floc formation from raw EPS components. Where PLS, HLS and UC are the protein-like substances, humic-like substances, unknown components and their complexes with metals are PLS-M, HLS-M and UC-M, respectively. Photo-flocculation could occur simultaneously along with photoinduced EPS (or DOM) in aqueous media, thereby forming complexes between free metals and newly formed DOM (photoproducts) that ultimately generate TEPs/flocs. During the end of the processes, humic-like substances are completely decomposed, and their ability to form floc/TEP is decreased. Protein-like substances of EPS predominantly participate in TEP/flocculation processes.

Based on the results, we obtained in this study, we propose a conceptual model for the formation of TEP/floc from the photoinduced EPS (Fig. 7). According to this model, the molecular compositions of EPS, when decomposed, can form new protein-like components. Such newly formed protein-like components can form complexes with free metals produced from the photodegradation of EPS and its molecular components. Metal-protein complexation is a well-known phenomenon in both water-based and biological systems^6, 9, 35–40^. Correspondingly, our proposed model is supported by earlier studies wherein proteins have been shown to form aggregates at low metal ion concentrations^37, 38, 41^. However, our model is partly inconsistent with the previous mechanism of photo-flocculation of bulk DOM first provided by Helms and his colleagues^8^, which is possibly attributed to not identifying the molecular composition of the DOM and its photic end-products. Helms and his colleagues suggested that photo-flocculation occurred via two or more pathways. They reported that initial flocculation of organic and inorganic material might not occur simultaneously. In asserting a mechanism, they also proposed that during “Phase I” organic matter flocculates but that Fe does not. During “Phase II” both organic matter and Fe flocculate. There is some lack of understanding of this mechanism that does not fit well with the current knowledge of photochemistry. For example, it is generally known that DOM, including EPS is photochemically decomposed, particularly in the case of humic substances (e.g., fulvic and humic acids) upon sunlight exposure in natural waters^34, 42–46^. This ultimately produces low-molecular-weight DOM^25^ and mineralisation of other products, such as CO_2_, DIC (dissolved CO_2_, H_2_CO_3_, HCO_3_^−^ and CO_3_^2−^), NH_4_^+^and PO_4_^3−^ ^9, 47, 48^. Such decompostion of DOM was not supported by the aforementioned mechanism. The second issue is that over 99% of dissolved Fe is strongly complexed with the functional groups of DOM in marine waters^49–51^. In essence, high-molecular-weight DOM including EPS, often exists as complexation with trace metals (M), such as, DOM-M or EPS-M in natural waters, which also did not appear in the aforementioned mechanism. FTIR and SEC analysis of TEP/flocs in our study demonstrated that TEP/flocs were composed of organic acid-linked structural phenomena which were protein dominant. Correspondingly, formation of protein-like or tyrosine-like substances (as identified from EEM-PARAFAC modelling) in irradiated samples is crucial for better understanding of this mechanism. As discussed earlier the protein-like substances can form complexes with metals. The third issue is that our time-lapse images of the EPS-to-TEP-size transformation showed that TEP/floc formation in aqueous solution is gradually increased from the 0 hour to the 58-hour time points.

### Proposed mechanisms of photo-flocculation

Considering the above mentioned issues, we suggest that a simultaneous process could link TEP/floc formation to the degradation of EPS (or DOM) in aqueous media, thereby forming complexes between free metals and newly formed DOM that ultimately generate TEP/flocs. In these mechanism, the initial complex between EPS and metal abbreviated as EPS-M upon light exposure can produce protein-like and humic-like substances and unknown components, any of which are considered to be complexed with metals in the solution (Eqs 1–2). Interestingly, EPS raw solution contained high contents of different trace metals, such as Fe (905.9 ppb), Zn (20.2 ppb), Al (5.6 ppb), Ni (25.3 ppb), Cr (7.1 ppb), As (70.1 ppb), Ba (30.4 ppb), Pb (0.8 ppb) and Cd (0.02 ppb) in this study and depending on the complexation capacity of the metals, they formed complexes with EPS. Such EPS-M complexation has been reported in earlier studies^6, 21, 41^. However, after 58 hours of irradiation, all humic-like substances were entirely decomposed (Fig. 4), while the remaining protein-like substances could complex with free metals, which could correspondingly form the TEP/floc. Because TEP/floc is composed of organic substances exhibiting various functional groups (−COOH, −C = O, −OH etc.) identified by FTIR spectra (Fig. 5A,B), they could be produced either via the complexation of PLS-M, HLS-M and UC-M produced during rearrangement of PLS, HLS and/or their photodegradation (Eq. 3). Where, PLS, HLS and UC are the protein-like substances, humic-like substances, unknown components and their complexes with metals are PLS-M, HLS-M and UC-M, respectively. Note that the humic-like substance was completely decomposed and it played a minor role in flocculation processes other than as an electron acceptor and donor^3, 5^. The protein like substance^3, 5^, unknown components and the unknown photo-products could be responsible for TEP/floc formation. Therefore, given EPS as autochthonous DOM and its complexations with trace metals (M) abbreviated as EPS-M, the possible detailed mechanistic sequence of TEP/floc formation by sunlight is as follows (Eqs 1–3):

1 $${\rm{EPS}}-{\rm{M}}+h{\rm{\upsilon }}\to {\rm{PLS}}+{\rm{HLS}}+{\rm{UC}}+{\rm{M}}$$

2 $${\rm{PLS}}+{\rm{HLS}}+{\rm{UC}}+{\rm{M}}+h{\rm{\upsilon }}\to {\rm{PLS}}-{\rm{M}}+{\rm{HLS}}-{\rm{M}}+{\rm{UC}}-{\rm{M}}$$

3 $$\begin{array}{c}{\rm{PLS}}-{\rm{M}}+{\rm{HLS}}-{\rm{M}}+{\rm{UC}}-{\rm{M}}+h{\rm{\upsilon }}\to {\rm{PLS}}-{\rm{M}}({\rm{TEP}}/{\rm{floc}})\\ \quad \quad \quad +{\rm{UC}}-{\rm{M}}({\rm{TEP}}/{\rm{floc}})+{\rm{photoproducts}}\end{array}$$

Similar reaction mechanisms are reported for DOM-M degradation, where Fe^3+^–DOM complexes are decomposed because of the rapid excitation of π-electron bonding upon light exposure^34, 44–46^. During photochemical flocculation of the DOM and iron to POM, approximately 87% of the iron was removed from the dissolved phase after 30 days. However, iron did not flocculate until a major fraction of the DOM was removed by photochemical degradation and flocculation (>10 days); thus, during the initial 10 days, there were sufficient organic ligands present or the pH was low enough to keep iron in solution^8^. Note that the Fe^3+^–DOM complex would be formed by the donation of electrons from O- or S-containing functional groups of DOM, mostly humic substances (fulvic and humic acids)^9^ to an outer unpaired *d*-orbital of Fe^3+^ (^25^Fe^2+^: 1*s* ^2^2*s* ^2^2*p* ^6^3*s* ^2^3*p* ^6^3*d* ^2^_xy_3*d* ^2^_xz_3*d* ^1^_yz_3*d* ^1^_×2–y2_3*d* ^1^_z2_4*S* ^0^) and Al^3+^ (1*s* ^2^2*s* ^2^2*p* ^6^3*d* ^3^3*S* ^0^3*p*_x_^0^3*p*_y_^0^3*p*_z_). Due to the presence of protein-like components in the sample irradiated for 58-hours, free trace metals preferentially form complexes with only newly formed protein-like substances (Fig. 4C), thereby forming metal ion complexation between protein-like or unknown DOM components and dissolved Fe or Al or colloids of EPS. Such metal-protein (or DOM molecules) complexation is reported in earlier studies^6, 9, 38, 40, 52^, thereby forming aggregation^8, 15, 37, 38, 53^. Metal-protein binding is often observed through C = O or C−N with the precipitation of tyrosine residues^38^. Although photo-flocculation of the EPS occurred throughout 58 hours of sunlight exposure, all the Fe and Al essentially remained in the solution because of the complete decomposition of the humic-like substances and either partial degradation or TEP/floc formation of protein-like substances. Because EPS is considered to be complexed with metals (e.g. Fe), it is either quickly recycled back into the dissolved phase by complexation with protein-like substances, or it photo-reduced to Fe (II), which was soluble in the experimental pH condition^8^. Rapid decomposition of EPS-M complexes is considered to take place due to a rapid excitation from its π-electron bonding system upon light exposure^9^. It can be noted that lectin-like proteins of EPS might be responsible for aggregation and floc formation^5, 51^.

This study confirms that EPS can form TEP and flocculates in the presence of sunlight. Increasing TEP concentrations can result in further significant accumulation of carbon-rich components that subsequently enhance sedimentation of POM^53^. TEP/floc thus plays an important role in recirculation as an additional source of nutrients, trace elements and DOM to the water column from deep sediment in the winter season, which is a result of vertical mixing in surface waters, particularly in lakes and oceanic environments^9, 54^. Finally, when considering the peak formation of TEP after 19-hours (2^nd^ day) of photo exposure (Fig. 1B), with its successive decrease in concentration from the 32 hours to 58 hours (3^rd^ to 5^th^ day). It is notable that total cumulative sunlight illuminated intensity was ~7216 MJ/m^2^ with frequency >0.43–1.50 × 10^6^ GHz on the second day and a total illuminated intensity of 7701 MJ/m^2^ (counting solar intensity at every minute) thereafter. These findings suggest that flocs/TEP can be formed and be stable at first 19-hours and then decompose successively. Such an assessment could be supported by the turbidity and other observations (Fig. 3A), for which the highest turbidity is observed after 19-hours and then decreases subsequently. *In-situ* experiments are important to further confirm the stability of flocs/TEP in lake or oceanic environments, which could offer foci for further research. In essence, the detection of fluorescent components using a combination of EEM-PARAFAC modelling is an important paradigm for better understanding TEP/floc formation from EPSs under the irradiation processes.

## Methods

### Collection of the microbial mat

Bosten Lake in Kuerle, Xinjiang Province, China, is an interesting ecosystem in so far as the salinity gradient flows from fresh to sub-saline. Microbial mat samples of alga-bacteria rich in EPS were collected from the pond west of Bosten Lake (N 42° 01.269′ and E 86° 47.293′ ) (Supplementary Fig. 1) on the 28^th^ of November 2014. The temperatures on the date of sample collection varied from (−4° to −12 °C). The frozen mat samples were collected in a container and immediately transported to the Xinijinag Institute of Ecology and Geography and preserved at −20 °C.

The remaining details for the experimental design for photo-flocculation, extraction and characterisation of EPS and all analytical methods are reported in the Supplementary Information.

## Electronic supplementary material

Supplementary Information
