# Supplementary material for: Photo-flocculation of microbial mat extracellular polymeric substances and their transformation into transparent exopolymer particles: Chemical and spectroscopic evidences
Source: Sci Rep. 2017 Aug 22;7:9074. doi: 10.1038/s41598-017-09066-8 (PMC5567378; doi:10.1038/s41598-017-09066-8)
Supplement: Supplementary file 1 — Supplementary Information [file 41598_2017_9066_MOESM1_ESM.pdf]

Supplementary Information

Photo-flocculation of microbial mat extracellular polymeric substances and their transformation into transparent exopolymer particles: Chemical and spectroscopic evidences

Mashura Shammi[1,2,6], Xiangliang Pan[1,5*], Khan M.G. Mostofa[3*], Daoyong Zhang[1], and Cong-Qiang Liu[4]

[1]Laboratory of Bioremediation, Department of Environmental Pollution and Process Control, Xinjiang Institute of Ecology and Geography, Chinese Academy of Sciences, Urumqi−830011, Xinjiang, PR China.
[2]Department of Environmental Sciences, Jahangirnagar University, Dhaka 1342, Bangladesh.
[3]Institute of Surface-Earth System Science, Tianjin University, 92 Weijin Road, Nankai District, Tianjin 300072, P.R. China.
[4]State Key Laboratory of Environmental Geochemistry, Institute of Geochemistry, Chinese Academy of Sciences, Guiyang 550002, Guizhou, P. R. China.
[5]College of Environment, Zhejiang University of Technology, Hangzhou 310014, Zhejiang, P.R. China
[6]University of Chinese Academy of Sciences, Beijing 100049, P.R. China

[*]**Corresponding author:** E−mail: xiangliangpan@163.com (XL Pan)
Phone: +861509906711 or Email: mostofa@tju.edu.cn; Phone: +8615122054195 (KMG Mostofa)

Contents of the file

Methods
Supplementary References
Supplementary Figure S1
Supplementary Figure S2
Supplementary Table S1
Supplementary Table S2

Methods

Extraction of EPS

The microbial mat samples were washed with MQ water at least 10 times and dispersed into suspension by a stirrer[1] followed by ultrasonication at 40 Watt in an ice bath for two minutes[2] which would not cause cell lysis[1,3]. The sonicated suspension was then centrifuged at 16,000 rpm, 4°C for 20 min[1]. The supernatants were filtered through a 0.22 µm polycarbonate filters, which were used throughout the experiment. The filtrate was used as the EPS sample[3]. The extracted EPS from microbial mat by ultrasonication[11] in this study generated whole EPS without significantly changing it. The EPS solution was immediately stored at ─20°C until further use.

Experimental design for photo−flocculation

Three sets of transparent Erlenmeyer flasks were filled with the EPS samples (150 ml) extracted from microbial mat collected from a saline pond west of Lake Bosten. The flasks were sealed with glass caps and Teflon tape, and they were kept in a water bath set up on the roof of the Xinjiang Institute of Ecology and Geography, Xinjiang, China. Tap water was continuously supplied to control the temperature. Samples of approximately 20 ml were taken each time interval from the flasks for further analysis. Sample collection time was fixed at 0, one, two, six hours (first day), 19 hours (second day), 32 hours (third day) and 58 hours (fifth day). Analytical measurements included changes in TEP, surface charge ζ− potential and particle size distribution, turbidity, pH, total protein and polysaccharides, dissolved organic carbon (DOC), FTIR, size exclusion chromatography (SEC) and EEM-PARAFAC modelling.

Characterisation for photo−flocculation

To examine the effects of solar radiation on extracted EPS transformation into TEP and floc particle, a modified Alcian blue assay was used for TEP measurement[4]. In addition, changes in surface charge ζ− potential and particle size distribution, turbidity, pH, changes in total protein and polysaccharide and dissolved organic carbon (DOC) were also measured. Moreover, FTIR and size exclusion chromatography (SEC)[5] along with changes in fluorescence properties by EEM-PARAFAC modelling were also used to measure EPS-flocculation. Fluorescence (excitation emission matrix, EEM) spectroscopy reveals the characteristic features of EEM spectra that provide useful information about biogeochemical cycling of a large proportion of the DOM pool, including a broad suite of unseen molecules that apparently follow the same gradients as fluorescent DOM (FDOM) in the environment[6-7]. Parallel factor (PARAFAC) modelling is extensively used on EEM spectra to isolate and quantify the individual fluorescence component signals in terms of fluorescence intensity[7-10].

Dissolved organic carbon (DOC) was measured by an Analytik Jena multi N/C 2100 (Germany). No filtration was conducted before DOC because EPS was filtration with 0.22-µm filter paper. Turbidity of the EPS solution was measured using a light−scattering photoelectric turbidity meter (HACH−2100P, Shanghai). pH was measured by a portable pH meter (metro ohm). Surface charge ζ− potential (−mV) and particle size distribution (PSD) were used to investigate flocculation of EPS by Zetasizer Nano ZS90 (Nano series, Malvern Instruments, UK). The samples were carefully filled into ζ− potential folded capillary cells (DTS1060) to avoid air bubble formation.

Measurement of photo intensity during experimental days

Cumulative hourly solar radiation intensity data (MJ/m^2) was taken from Xinjiang Uygur Autonomous Region Meteorological Bureau (Supplementary Table 1). The total cumulative sunlight intensity was varied from the first day (32 at 5-6 am, 448 at 11-12 am and 11 MJ/m^2 at 18-19 pm, respectively) to the fifth day (6 at 5-6 am, 222 at 15-16 pm and 11 MJ/m^2 at 18-19 pm, respectively) with a total of 14917 MJ/m^2 counting at every minute during the five days period. The photo active radiation (PAR) value of the light was measured with a Quantam Meter (Model ESM−Q1, Ecotron Scientific Inc.) in µmol photons $m^{-2}s^{-1}$. Daytime UV intensity and air temperature were drawn from the website (website: http://www.qx121.cn/qxfw/ASP/wlmqzwx.asp). PAR intensity varied from 1041 µmol photons m^{-2} s^{-1} at 10:00 am to 25 µmol photons m^{-2} s^{-1} at 21:00 pm in the afternoon which reached its peak at an average 1985 µmol photons m^{-2} s^{-1} at the noon during the five days experimental period. This shows that natural sunlight intensity was approximately 10 to 15 times stronger used in this study than earlier study (Supplementary Figure 2a).

For every experimental time interval before data collection and the finishing time water bath temperature was measured with thermometer which varied in the start from 22ºC and rise up to 30ºC which was maintained by a continuous water flow (Supplementary Figure 2b-c). Note that temperature variation in the water bath from 22 to up to 30 ºC is similar phenomenon to diurnal or summer-winter variation in surface waters. Moreover, recent study shows that an increase in temperature of 10°C to 15°C produced an approximately 15% increase in particulate organic carbon photodegradation and a 20% increase in DOC photodegradation[11].

Dark control experiment

A control experiment on EPS flocculation was performed in a dark room temperature, wherein flasks were wrapped with aluminium foils. EPS samples were collected at 0 hours, 32 hours (three days) and 58 hours (five days), at similar times of sunlight exposure as upon sample collection. Only daylight exposure hours (12 hours per day) were counted, to the exclusion of night hours. DOC, TEP, ξ−potential, PSD, pH and turbidity of EPS samples were measured under dark control, which showed no significant changes in the respective measurement parameters under dark control during the experimental time period (Supplementary Table 2).

Calibration protocol for $TEP_{0.22\,\mu m}$ with Gum Xanthan

The modified Alcian blue assay for TEP measurement[4] was capable of determining both TEP and their precursors in a single analysis measured via UV spectrometer (UV−2550, SHIMADZU) at a wavelength of 610 nm. There is a direct relationship between the amount of Alcian blue removed and the TEP concentrations. Two sets of solutions with different xanthan gum concentrations (0, 2.5, 5, 8, 10 µg/mL) were prepared for the establishment of a calibration curve (Fig. 1a). The TEP concentration was expressed as the equivalent of xanthan gum concentration. The xanthan gum calibration curve exhibited a linear relationship, with an R-squared value of 0.99, within the concentration range of 0–10 µg Xanthan gum (GX) equivalent/mL.

Microscopic time lapse image collection of EPS to TEP transformation

Five-millilitre EPS samples were taken in a 10-mL tube and dyed with 0.50 mL Alcian blue dye; these mixtures were vortexed for 1 minute followed by standing in the dark for 30 minutes. 0.50 mL Alcian blue dyed EPS were placed on the slide. Images were collected with an Olympus

BX51TRF equipped with PixeLINK 134 color camera (Kodak) and Linksys32 software at 5X magnification.

FTIR analysis of EPS
EPS samples were oven dried at 60°C and then the dried samples were mixed with solid KBr in the ratio of 1:100. The KBr–EPS pellets of 13 mm diameter of the mixture were prepared at 8.103 kg cm^{-2} pressure. The FTIR spectra of the EPS samples were recorded with a FTIR spectrophotometer (Bruker Tensor 27) and analysed with OPUS software 5.5. For each interferogram 256 scans were taken in the 400–4000 $cm^{-1}$with a resolution of 2cm^{-1}.

Sample preparation for FTIR analysis of TEP
After the sunlight treatment of EPS, the 58 hours of sunlight treated samples were filtered on 0.22 μm polycarbonate filter paper. The filter paper was carefully placed on a 4 mL tube and rinsed with MQ water. The tubes were then placed in a shaker for 10 minutes followed by freeze dry at −70°C and scanned as mentioned above.

Size exclusion chromatography (SEC)
Protein molecular weight of EPS was qualitatively determined. A Merck Hitachi LA Chrom Chromatograph equipped with a L2200 auto sampler, a L2130 quaternary pump, a L2300 column oven interface and a L2455 diode array UV detector (200-600 nm), with software version LaChrom 890−8800−12 was used. An Amersham Biosciences column, Superdex 200 10/300 GL, was used (resolving range from 10 to 600 kDa). Data processing was done with EZchrom Elite. The mobile phase recommended elsewhere[5]. Injection volume was 80 μL EPS. Molecular weight 14.4 kDa – 94.7 kDa was used for qualitative protein analysis which contained protein markers of 94 kDa, 66.2 kDa, 45 kDa, 33 kDa, 26 kDa, 20 kDa and 14.4 kDa (TIANGEN MP102, Senggong Biotech). The entire 3 injections obtained 5 peak positions in the chromatogram according to the standard markers in retention time 18.27 to 22.08 minute, 29.42 to 32.38 minute, 40.42 to 41.83 minute, 50.58 to 52.41 minute and 58.20 to 62.01 minute, respectively. After obtaining the standard peak the sample injections were run original EPS and EPS treated with sunlight.

EEM-PARAFAC modelling
The fluorescence (excitation−emission matrix, EEM) spectra of the EPS solution were recorded with a fluorescence spectrophotometer (F−4500, HITACHI, Japan) in scan mode with a 700−voltage xenon lamp at room temperature. The EEM spectra were collected at 5 nm increments over an excitation range of 200–400 nm, with an emission range of 250–500 nm by every 1nm. The excitation and emission slits were set to 5 nm of band−pass, respectively. The scan speed was 1200 nm min^{-1}. All the cuvettes before analysis were rinsed and ultra−sonicated using 5% (w/w) nitric acid solution. The Milli−Q water blank was subtracted from the sample's EEM spectra. Fluorescence data were interpreted and its components were identified using parallel factor (PARAFAC) modelling, which is a three-way multivariate analysis that is widely used currently[7-10]. This study conducted three sets of PARAFAC modelling on the EPS sample EEM spectra. In the first set, original raw EPS sample before irradiation and its diluted samples with Milli-Q pure water were processed for three component analyses which covered approximately 94% of the variations in EEM values. PARAFAC modelling for sunlight irradiated samples was performed on the EPS samples for short term irradiation (one to six hours)

and long term irradiation (19 hours to 58 hours), which covered approximately 97% and 85% variation of the EEM data of the samples for three component analyses, respectively.

EPS protein and polysaccharide analysis

The quantities of polysaccharides in EPS samples were determined by the phenol–sulfuric acid method by Dubois et al.[12] where glucose was used as a standard. Absorbance was measured at 488 nm against blank to determine the polysaccharide in this study. The protein content of the EPS was determined using modified Lowry procedure for room temperature[13] recommended by Frolund et al.[14] for microbial EPS using bovine serum albumin (BSA) as a standard (Sengon Biotech, Shanghai). In protein analysis, absorbance of the samples was measured at 750 nm against blank. A calibration curve was prepared before each analysis. A UV spectrophotometer (UV−2550, SHIMADZU) was used in both measurements.

Trace element analysis

To measure the different trace metals such as Fe, Zn, Al, Ni, Cr, As, Ba, Pb and Cd in the original extracted EPS sample, a Perkin-Elmer Elan 6100 ICP-MS (Inductively Coupled Plasma Mass Spectrometer) was used along with a Perkin-Elmer AS 93 PLUS autosampler. EPS sample was filtered with 0.45 µm polycarbonate filter and was not acidified. Standard curves were obtained containing known concentrations of each heavy metal that were diluted with Milli-Q pure water. All reagents were of analytical grade. The standard error was determined to be less than 5%. All glassware used for trace element analysis were soaked in 25% HNO_3 for 24 hours, rinsed, and then dried.

Statistical analysis

All EPS characterizations and measurements were performed thrice and a standard error was calculated. All the data and processed spectra were exported to Origin 8.0 (OriginLab, USA) for preparation of graphs.

Supplementary References

1. Liu, H. and Fang, H. H. P. Extraction of extracellular polymeric substances (EPS) of sludges. J. Biotechnol. **95**, 249−256 (2002).
2. Comte, S., Guibaud, G. and Baudu, M. Relations between extraction protocols for activated sludge extracellular polymeric substances (EPS) and EPS complexation properties. Enzyme Microb. Technol. **38**, 237−245 (2006).
3. Pan, X *et al.* A comparison of five extraction methods for extracellular polymeric substances (EPS) from biofilm by using three−dimensional excitation−emission matrix (3DEEM) fluorescence spectroscopy. Water SA. **36**, 111−116 (2010).
4. Arruda Fatibello, S. H. S., Henriques Vieira, A. A., & Fatibello-Filho, O. A rapid spectrophotometric method for the determination of transparent exopolymer particles (TEP) in freshwater. Talanta **62**, 81-85 (2004).
5. Simon, S. *et al*. Evaluation of size exclusion chromatography (SEC) for the characterization of extracellular polymeric substances (EPS) in anaerobic granular sludges. Bioresour. Technol. **100**, 6258−6268 (2009).

6. Moran, M. A., Sheldon, W. M., Jr. & Zepp, R. G. Carbon loss and optical property changes during long-term photochemical and biological degradation of estuarine dissolved organic matter. Limnol. Oceanogr. **45**, 1254–1264 (2000).
7. Mostofa, K. M. G, Yoshioka, T., Mottaleb, A. & Vione, D. Photobiogeochemistry of organic matter: Principles and practices in water environments. (Springer, 2013).
8. Stubbins, A. *et al*. What's in an EEM? Molecular signatures associated with dissolved organic fluorescence in boreal Canada. Environ. Sci. Technol. **48**, 10598-10606 (2014).
9. Borisover, M., Laor, Y., Parparov, A., Bukhanovsky, N. & Lado, M. Spatial and seasonal patterns of fluorescent organic matter in Lake Kinneret (Sea of Galilee) and its catchment basin. Water Res. **43**, 3104–3116 (2009).
10. Stedmon, C. A., Markager, S., & Bro, R. Tracing dissolved organic matter in aquatic environments using a new approach to fluorescence spectroscopy. Mar. Chem. **82**, 239-254 (2003).
11. Porcal. P., Dillon, P.J., Molot, L.A. Temperature dependence of photodegradation of dissolved organic matter to dissolved inorganic carbon and particulate organic carbon. PLoS ONE 10, e0128884 (2015).
12. Dubois, M. *et al*. Colorimetric Method for Determination of Sugars and Related Substances. Anal. Chem. **28**, 350−356 (1956).
13. Lowry, OH, NJ Rosbrough, AL Farr, and RJ Randall. *J. Biol. Chem.* **193**, 265 (1951).
14. Frølund, B., Palmgren, R., Keiding, K. and Nielsen, P.H. Extraction of extracellular polymers from activated sludge using a cation exchange resin. Water Res. **30**, 1749−1758 (1996).

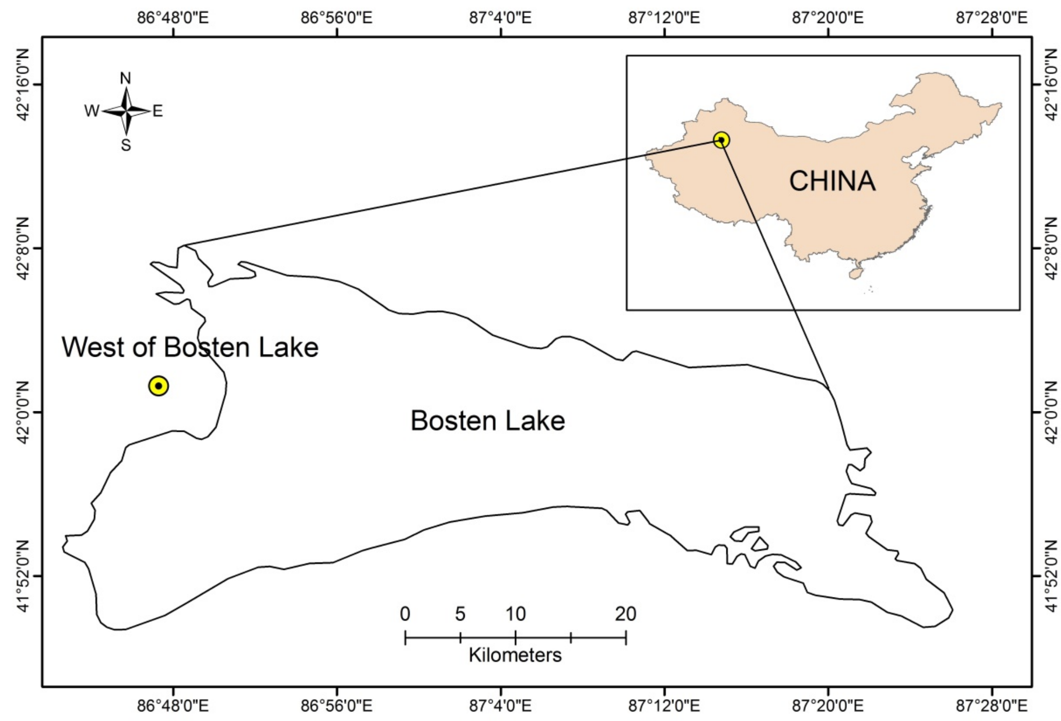

Supplementary Figure S1. Sampling site of saline pond near Bosten Lake. Sampling point shown in yellow mark. Garmin GPSMAP 60CSx was used to record global positioning system (GPS) data by sampling point during field work. The source of base map of China and lake base map was taken from Google Earth. The inland water shape file was used in this map which included separate files for line and area features for rivers, canals and lakes in vector format. Sources of the shape files taken from Digital Chart of the World from Diva GIS (website: http://diva-gis.org/gdata). Datum used in this map preparation was The World Geodetic System (WGS 1984) under the Geographic coordinate system (GCS) in degree units. The final map was produced by ArcGIS 10.3 (Esri, USA) (website: http://www.esri.com).

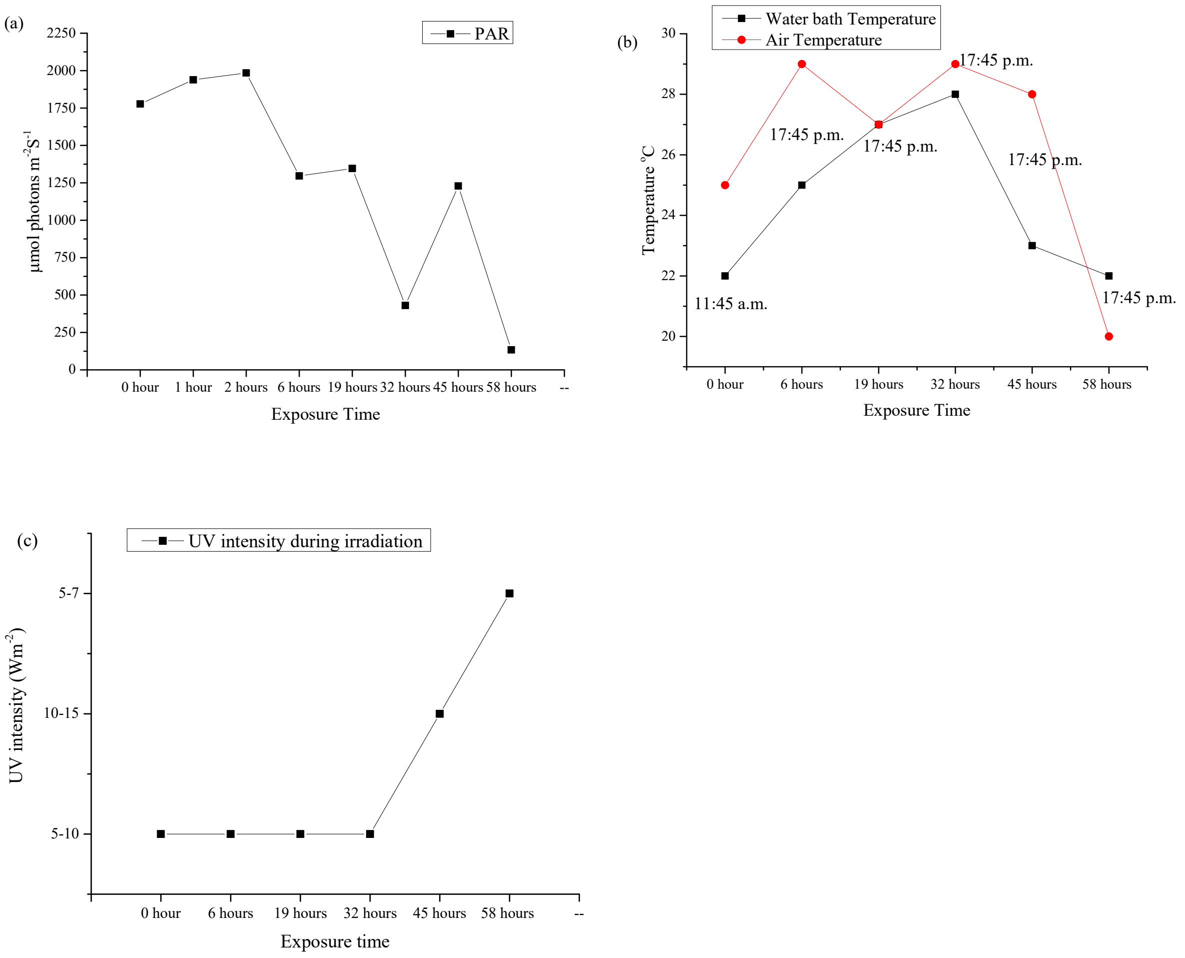

Supplementary Figure S2. Experimental condition during the experimental time (a) Photo active radiation; (b) air and water bath temperature variation of the experimental time; and (c) UV intensity of the day. Only daylight exposure hours (12 hours per day) were counted, to the exclusion of night hours.

Supplementary Table S1. Cumulative hourly solar radiation intensity data (MJ/m^2) during the experimental days over 24 hour cycle

Hours	00-01	01-02	02-03	03-04	04-05	05-06	06-07	07-08	08-09	09-10	10-11	11-12	12-13	13-14	14-15	15-16	16-17	17-18	18-19	19-20	20-21	21-22	22-23	23-00
2015-5-8	...	...	...	...	1	32	120	205	297	370	421	448	447	401	327	313	88	65	11	0	...	...	...	...
2015-5-9	...	...	...	...	0	0	86	214	299	369	418	439	442	411	362	292	204	111	24	0	...	...	...	...
2015-5-10	...	...	...	...	1	38	114	210	291	368	419	434	432	393	315	160	92	51	19	0	...	...	...	...
2015-5-11	...	...	...	...	1	32	116	208	287	353	401	427	406	387	324	188	120	88	21	0	...	...	...	...
2015-5-12	...	...	...	...	1	6	12	39	0	0	0	94	89	182	184	222	126	42	11	0	...	...	...	...

Supplementary Table S2. Some flocculation parameters measured in dark room incubated EPS (25°C)

Time	DOC (mgL^{-1})	TEP (μg/mL GX)	ξ−potential (-mV)	PSD (dnm)	pH	Turbidity (NTU)
0 hours	86.03±0.1	0.67±0.1	-15.53±1.8	1003.6±8	8.7±0.01	16.8±0.3
30 hours	86.70±0.5	0.56 ±0.02	-14.4±1.8	809.27±4	8.62±0.1	21.6±0.1
54 hours	85.32±0.1	0.49±0.05	-14.05±0.3	951.4±13	8.60±0.02	24.2±0.05
